# Genome Sequence of the Oocydin A-Producing Rhizobacterium Serratia plymuthica 4Rx5

**DOI:** 10.1128/MRA.00997-18

**Published:** 2018-09-20

**Authors:** Miguel A. Matilla, Zulema Udaondo, George P. C. Salmond

**Affiliations:** a Department of Biochemistry, University of Cambridge, Cambridge, United Kingdom; b Department of Environmental Protection, Estación Experimental del Zaidín, Consejo Superior de Investigaciones Científicas, Granada, Spain; c Department of Biomedical Informatics, University of Arkansas for Medical Sciences, Little Rock, Arkansas, USA; University of Delaware

## Abstract

Serratia plymuthica 4Rx5 was isolated from the rhizosphere of oilseed rape due to its antagonistic properties against plant-pathogenic fungi. The strain 4Rx5 produces the antifungal and antioomycete haterumalide, oocydin A. Analysis of its genome revealed the presence of various gene clusters putatively involved in the biosynthesis of additional secondary metabolites.

## Abstract (precis)

Serratia plymuthica 4Rx5 was isolated from the rhizosphere of oilseed rape due to its antagonistic properties against plant-pathogenic fungi. The strain 4Rx5 produces the antifungal and antioomycete haterumalide, oocydin A.

## ANNOUNCEMENT

Serratia plymuthica strains are frequently isolated from the rhizosphere of agriculturally relevant crops and are considered efficient biocontrol agents (1, 2). Their biocontrol properties have been associated with the production of antibiotics (1–6), hydrolytic enzymes (1, 2), and their ability to trigger systemic resistance (2, 7).

Serratia plymuthica 4Rx5 was originally isolated by Berg and coworkers (1) from the rhizosphere of oilseed rape after screening for bacteria that showed hydrolytic activities and antagonism against phytopathogenic fungi. More recently, strain 4Rx5 has been used as a model bacterium for the investigation of biosynthesis and regulation of the antifungal oocydin A (8). In contrast to other oocydin A-producing strains, the biosynthesis of this polyketide in 4Rx5 was shown to be regulated by an *N*-acyl-l-homoserine lactone-based quorum-sensing-system (8). Additionally, 4Rx5 was shown to produce chitinases and proteases (1) and siderophores (M. A. Matilla and G. P. C. Salmond, unpublished data).

The genomic DNA of 4Rx5 was extracted from stationary-phase cells grown in lysogeny broth (9) using a Qiagen DNeasy kit. A single-end shotgun library for 454 pyrosequencing was prepared using a Roche GS FLX Titanium rapid library preparation kit and was run on a picotiter plate for a Roche Applied Science Genome Sequencer FLX instrument according to the manufacturer’s specifications. Read quality was monitored with the inclusion of control reads and using the 454 sequencing system software package v2.6 (Roche) using default settings. The resulting 319,495 reads were *de novo* assembled using Newbler v2.6 with default parameters. An approximately 25× coverage of the estimated genome size was obtained, and the assembly resulted in 20 contigs larger than 1,000 bp. The largest contig was 1,704,970 bp, and the *N*_50_ contig size was 511,280 bp. The genome was automatically annotated using NCBI Prokaryotic Genome Annotation Pipeline v4.2 (10).

The draft genome sequence of 4Rx5 comprises 5,367,478 bp with an overall G+C content of 54.7%. Automated genome annotation predicted 4,870 protein-coding sequences, 75 pseudogenes, 1 CRISPR array, 6 rRNAs, 71 tRNA genes, and 9 noncoding RNAs. In addition to the polyketide synthase biosynthetic cluster responsible for the production of oocydin A (5), bioinformatic analyses using antiSMASH (11) predicted 4 additional uncharacterized gene clusters putatively involved in the production of polyketides and nonribosomal peptides. Scrutiny of the genome also revealed the presence of a biosynthetic cluster responsible for production of the antifungal metabolite pyrrolnitrin (12). Genome comparison analyses showed that the genome of 4Rx5 is highly similar to that of Serratia plymuthica 4Rx13 (13). The strain 4Rx13 produces a broad range of volatile organic compounds (VOCs) (13–15), some of them possessing antifungal properties (14). The bicyclic terpene sodorifen was the major VOC emitted by 4Rx13 (15), and the sodorifen gene cluster was identified in the genome of 4Rx5. Altogether, our results highlight the potential of 4Rx5 for the biocontrol of phytopathogenic fungi and oomycetes. Further research will elucidate the spectrum of secondary metabolites produced by this bacterium.

### Data availability.

The sequences obtained by this whole-genome shotgun project have been deposited in DDBJ/EMBL/GenBank under the accession number PESE00000000.
